# Effect of St. John's Wort (*Hypericum perforatum*) treatment on restraint stress-induced behavioral and biochemical alteration in mice

**DOI:** 10.1186/1472-6882-10-18

**Published:** 2010-05-07

**Authors:** Anil Kumar, Ruchika Garg, Atish K Prakash

**Affiliations:** 1 Pharmacology Division, UGC Centre of Advance Study (UGC-CAS), University Institute of Pharmaceutical Sciences, Panjab University, Chandigarh-160014, India

## Abstract

**Background:**

A stressful stimulus is a crucial determinant of health and disease. Antidepressants are used to manage stress and their related effects. The present study was designed to investigate the effect of St. John's Wort (*Hypericum perforatum*) in restraint stress-induced behavioral and biochemical alterations in mice.

**Methods:**

Animals were immobilized for a period of 6 hr. St. John's Wort (50 and 100 mg/kg) was administered 30 minutes before the animals were subjecting to acute immobilized stress. Various behavioral tests parameters for anxiety, locomotor activity and nociceptive threshold were assessed followed by biochemical assessments (malondialdehyde level, glutathione, catalase, nitrite and protein) subsequently.

**Results:**

6-hr acute restraint stress caused severe anxiety like behavior, antinociception and impaired locomotor activity as compared to unstressed animals. Biochemical analyses revealed an increase in malondialdehyde, nitrites concentration, depletion of reduced glutathione and catalase activity as compared to unstressed animal brain. Five days St. John's Wort treatment in a dose of 50 mg/kg and 100 mg/kg significantly attenuated restraint stress-induced behavioral (improved locomotor activity, reduced tail flick latency and antianxiety like effect) and oxidative damage as compared to control (restraint stress).

**Conclusion:**

Present study highlights the modest activity of St. John's Wort against acute restraint stress induced modification.

## Background

Stress is a crucial determinant for maintenance of health and disease [1,2]. Stress either due to internal or external stimuli disturbs physiological homeostasis and causes neurobehavioral alteration [3,4]. There are various neuropsychiatric problems such as anxiety, cognitive dysfunction, depression etc, are generally associated with stress. Stress induces changes in emotional behavior, anxiety like state [5] that are associated with oxidative damage i.e. free radical damage [1,2]. Acute restraint stress stimulates numerous cellular cascade that lead to increase ROS production [6,7]. The central nervous system is especially vulnerable to free radical damage because of brain's high oxygen consumption, abundant lipid content and relative paucity of antioxidant enzymes [8]. Immobilization stress has also been reported to induce 2-3 fold higher rise of plasma cortisol level. Increase cortisol level has been linked with anxiety like behavior [9]. It has been reported that stress triggers the motor alteration in different animal models [10]. Previous studies have also demonstrated that various chronic stress triggers hyperalgesia and allodynia [10,11]. Recent studies demonstrated that restraint stress produces antinociception which is relevant to numerous painful pathologies, such as fibromyalgia (FM), characterized by diffuse muscular pain (hyperalgesia) and/or tenderness (allodynia). This contention supported by the findings that restraint stress increases pain threshold in hot-plate test [13]. The central nucleus of amygdala (CeA) is important in fear conditioning and in modulating affective response to stress [14-17].

St. John's Wort (*Hypericum perforatum*) is well known antidepressant herbal remedy contains hypericin, pseudohypericin, hyperforin, quercitine and quercitrin as one of the major active constituents [18]. SJW is widely used in the treatment of depression in many countries and represented as an accepted alternative to synthetic antidepressants or behavioral therapy, particularly for mild to moderate depression [19-22]. Recently, antidepressants have been reported to have neuroprotective effect and antioxidant like effect against immobilization stress [45]. However, their exact status in stressful conditions is still not clear so far.

With this background, the present study was designed to investigate protective effect of St. John's Wort in acute restraint-induced certain behavioral and biochemical modification.

## Methods

### Animals

Male Albino mice (Laca strain) weighing between 22-30 g bred in Central Animal House facility of the Panjab University, Chandigarh, India were used. The animals were housed under standard laboratory conditions and maintained on a 12 h light/dark cycle and had free access to food and water. Animals were acclimatized to laboratory conditions before the experiment. Each group consists of minimal 5 animals. All the experiments were carried out between 9:00 and 17:00. The experimental protocols were approved by Institutional Animal Ethics Committee of Panjab University, Chandigarh, India and conducted according to the Indian National Science Academy Guidelines for the use and care of experimental animals.

### Drugs and treatment schedule

**Figure 1:**
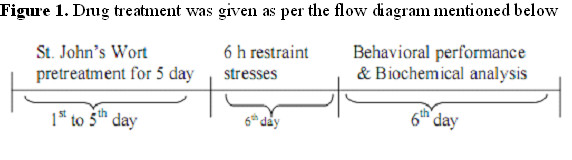


St. John's Wort (*Hypericum perforatu*) extract was provided by Dabur, Gaziabad, India as gift product. The extract was water soluble. Before administration, the extract was freshly prepared with distilled water. St. John's Wort (50 and 100 mg/kg, i.p.) were administered for five days and animals were subjected to restraint stress on 6^th ^day (Figure 1). The doses were selected based on our previous study [23].

### Restraint stress

Animals were restraint for 6-hr by taping all the four limbs on a board after putting them on their backs using zinc oxide hospital tape. Release was affected by unraveling the tape after moistening with acetone in order to minimize pain or discomfort. In unstressed group, the mice were kept in animal cage with soft bedding in the experimental room [24].

### Behavioral assessments

#### Measurement of antinociception

The nociceptive threshold was determined as the tail flick latency elicited in response to radiant heat [25]. Baseline latencies to tail flick withdrawal from the radiant heat source (3-5s) were established. A cut-off time of 10s was maintained to prevent any injury on the tail [26].

### Measurement of locomotor activity

Animal was kept in actophotometer for first 3 minutes before making actual recording in actophotometer for a period of 5 min. The apparatus was placed in a darkened light sound attenuated and ventilated testing room. Each animal was observed over a period of 5 min in a square (30 cm) closed arena equipped with infrared light sensitive photocells using digital photoactometer and values expressed as counts per 5 min [27].

### Measurement of anxiety: Mirror Chamber Test

The mirror chamber consisted of a wooden chamber having a mirror cube enclosed within it. The container box was (40 × 40 × 30.5) cm. Animal was placed at the distal corner of the mirror chamber at the beginning of the test. During the 5 min test session, following parameters were noted- a) latency to enter the mirror chamber, b) average time spent per entry in mirror chamber. An anxiogenic response was defined as decreased the number of entries and time spent in the mirror chamber [26].

### Biochemical parameters

All the animals were sacrificed by decapitation on the same day immediately after behavioral assessments. The brains were removed, rinsed in isotonic saline and weighed. A 10% (w/v) tissue homogenates were prepared with 0.1 M phosphate buffer (*p*H 7.4). The post nuclear fractions were obtained by centrifugation of the homogenate at 12000 × g for 20 min at 4°C.

### Lipid peroxidation assay

The quantitative measurement of lipid peroxidation in the whole brain was assessed as per method of Wills [28]. The amount of malondialdehyde formed was measured by the reaction with thiobarbituric acid at 532 nm using Perkin Elmer lambda 20 spectrophotometer. The results were expressed as nmol of malondialdehyde per mg protein using the molar extinction coefficient of chromophore (1.56 × 10^5 ^M^-1 ^cm^-1^).

### Estimation of reduced glutathione

Reduced glutathione (GSH) in the brain was estimated according to the method of Ellman [29]. A 1.0 ml of homogenate was precipitated with 1.0 ml of 4% sulfosalicylic acid by keeping the mixture at 4°C for 1 h and samples were immediately centrifuged at 1200 × g for 15 min at 4°C. The assay mixture contains 0.1 ml of supernatant, 2.7 ml of phosphate buffer of *p*H 8.0 and 0.2 ml of 0.01 M-dithiobisnitrobenzoic acid (DTNB). The absorbance of the reaction product was immediately measured at 412 nm using a Perkin Elmer lambda 20 spectrophotometer. The results were expressed as micromole GSH per mg protein.

### Nitrite estimation

Nitrite is the stable end product of nitric oxide (NO) in living system. Accumulation of nitrite was measured in cell free supernatants from brain homogenates by spectrophotometer assay based on Greiss reagent 15 (1% sulphanilamide/0.1% naphthylethylenediamine dihydrochloride/2.5% phosphoric acid) and incubated at room temperature for 10 min to yield a chromophore. Absorbance was measured at 543 nm spectrophotometrically. The nitrite concentration was calculated from a standard curve using sodium nitrite as standard and expressed as micro molar nitrite per ml homogenate [30].

### Protein estimation

The protein content was measured according to the method of lowry using bovine serum albumin as standard [31].

### Catalase estimation

Catalase activity was assayed by the method of Luck [32], wherein the breakdown of hydrogen peroxide (H_2_O_2_) was measured at 240 nm. Briefly, the assay mixture consisted of 3 ml of H_2_O_2 _phosphate buffer, 0.05 ml of supernatant of tissue homogenate (10%), and the change in absorbance was recorded at 240 nm. The results were expressed as micromole H_2_O_2 _decomposed per mg of protein/min.

### Statistical Analysis

All the values are expressed as mean ± SEM. The data were analyzed by using one way analysis of variance followed by Tukey's test. P < 0.05 was considered statistically significant.

## Results

### Behavioral measurements (Locomotor, anxiety and analgesic activity)

The naïve animals showed the consistent stable locomotor activity and anxiety like behavior and antinociception effect. 6-hr acute restraint stress significantly reduced locomotor activity (as indicated by decreased ambulatory movements), anxiety-like behaviors (delayed latency to enter in mirror chamber, decreased number of entries and time spent in the mirror chamber) and increased tail flick latency (antinociceptive like effect for first few minutes) as compared to unstressed (naïve) group (P < 0.05) (Table 1). Five days St. John's Wort (50 and 100 mg/kg) treatment significantly caused anti-anxiety-like behavior (shortened time latency to enter in mirror chamber, increased average time spent per entry in mirror chamber) and improved locomotor activity (increased ambulatory movements) as compared to control (restraint stress) animals. However, St. John's Wort treatment did not restore the performances of stressed mice to the levels of the naive group. However, tail flick latency was not influenced significantly as compared to control (restraint stress) animals (P < 0.05). Besides, St. John's Wort (50 and 100 mg/kg) treatment significantly produced analgesic effect in unstressed group as compared to naïve (P < 0.05) (Table 1).

**Table 1:** Effect of St. John's Wort on behavior alterations in unstressed and immobilization stressed animals

Treatment (mg/kg)	Locomotor activity test	Tail flick test	Mirror chamber test	
	(Counts/5 min)	Tail flick latency (Sec)	Latency to enter mirror chamber (sec)	Average time spent per entry(sec)
Naïve	232 ± 1.32	1.75 ± 0.10	43.0 ± 1.08	48.75 ± 1.65
SJW(50)	239 ± 4.2	4.0 ± 1.5^a^	37.6 ± 3.4	46.32 ± 2.8
SJW (100)	243 ± 6.9^a^	5.0 ± 2.8^a^	34.6 ± 4.6	44.3 ± 1.6
Control (RS)	48.75 ± 1.65^a^	9.25 ± 0.19^a^	127 ± 1.95^a^	12.25 ± 0.85^a^
SJW(50)+ RS	105.25 ± 2.0^b^	8.15 ± 0.45^a, NS^	101 ± 2.3^b^	21.5 ± 0.83^b^
SJW (100)+RS	125.25 ± 1.8^c^	7.82 ± 0.29^a, NS^	78 ± 2.8^c^	33.75 ± 0.76^c^

Valu0es are expressed as Mean ± SEM. ^a^P < 0.05 as compared to naïve. ^b^P < 0.05 as compared to control. ^c^P < 0.05 as compared to St. John's Wort (50 mg/kg). (One-Way ANOVA followed by Tukey's test). NS = Not significant, RS = Restraint Stress, SJW = St. John's Wort

### Biochemical measurements

6-hr acute restraint stress significantly increased malondialdehyde, nitrite concentration, depleted reduced glutathione and catalase activity as compared to unstressed (naïve) animals (Table 2). Five days with St. John's Wort (50 and 100 mg/kg) treatment significantly attenuated the rise in malondialdehyde, nitrite concentration and caused restoration of GSH and catalase activity as compared to the control (restraint stress) group (P < 0.05) (Table 2).

**Table 2:** Effect of St. John's Wort on biochemical alterations on unstressed and immobilization stressed animals

Treatment (mg/kg)	LPO (moles of MDA/mgpr)	GSH (μmol of GSH/mgpr)	Nitrite (μg/ml)	Catalase (μmol of H_2_O_2_/min/mgpr)
Naïve	0.112 ± 0.002	0.091 ± 0.002	254.5 ± 2.62	0.807 ± 0.003
S JW (50)	0.102 ± 0.002^NS^	0.094 ± 0.003^NS^	250.1 ± 1.58^NS^	0.809 ± 0.002^NS^
S JW (100)	0.100 ± 0.003^NS^	0.096 ± 0.002^NS^	249.5 ± 2.62^NS^	0.810 ± 0.002^NS^
Control (RS)	0.623 ± 0.006^a^	0.011 ± 0.0007^a^	653.5 ± 2.66^a^	0.140 ± 0.001^a^
S JW (50)+RS	0.340 ± 0.009^b^	0.034 ± 0.001^b^	501 ± 2.3^b^	0.300 ± 0.003^b^
S JW (100)+RS	0.190 ± 0.006^c^	0.064 ± 0.002^c^	394.75 ± 1.8^c^	0.465 ± 0.006^c^

Values are expressed as Mean ± SEM. ^a^P < 0.05 as compared to naïve. ^b^P < 0.05 as compared to control. ^c^P < 0.05 as compared to St. John's Wort (50 mg/kg). (One-Way ANOVA followed by Tukey's test) NS = Not significant, RS = Restraint Stress, SJW = St. John's Wort, LPO = lipid peroxidation, MDA = malondialdehyde, GSH = reduced glutathione, mgpr = milligram protein

## Discussion

The ability of the biological system to cope stressful condition plays a crucial role in the body [33]. Stress activates hypothalamus - pituitary - adrenal axis (HPA) axis and influences several neurological function at both central and peripheral level. Any kind of stress influences brain functions by causing long term changes in the multiple neural systems [34,35]. Restraint stress has been reported to influence motor activity, caused pain perception [37] anxiety like behavior [36], and depression-like behaviors [38] in the animals. In the present study, 6-hr restraint stress significantly caused anxiety like behavior, impaired motor activity and antinocieption, indicating stress induced neurobehavioral alterations. Stress has already been reported to alter neurobehavioral in both acute as well as chronic stress [38]. Marked behavioral changes might be due to alteration in the brain regions controlling motor activity and anxiety like behavior. Impaired motor activity could be due to stress induced depression. Further, St. John's Wort treatment for five days significantly improved behavior alterations, suggesting its neuroprotective effect against stressful conditions. Antidepressants have been reported to alleviate stress and stress related effects [39,40]. Recently, neuroprotective effects of antidepressants have been reported [45]. However, scientific mechanistic explanations of their clinical efficacy for treatment of depression are not been fully understood so far. Present study further suggests its therapeutic potential against these stress related altered behavioral states. Further, five days St. John's Wort treatment did not influence significantly antinociceptive effect. This might be due to its own analgesic effect, antianxiety and improved locomotor activity. In the present study, St. John's Wort treatment significantly produced analgesic effect. Antidepressant drugs have been widely used for many years to treat pain and related states, despite unclear rationale of their clinical use [45]. Their mechanisms of action (noradrenergic, serotonergic, opioids), focusing on central and peripheral analgesic actions are still not clear.

Oxidative stress causes cellular damage and accelerates neuro-degeneration by inducing the reactive oxygen species (ROS) that oxidize vital cellular components such as lipids, proteins and DNA [41,42]. In the present study, 6-hr immobilized stress caused significantly oxidative damage as indicated by raise in lipid peroxidation, nitrite concentration and depletes reduced GSH and catalase activity. Tsuboi et al reported an increased oxidative damage and weak antioxidant defense events are implicated in major depression [43]. In the present study, St. John's Wort treatment significantly attenuated lipid peroxidation, nitrite concentration and partially restored GSH and catalase activity suggesting its antioxidant like effect. Supporting to our study, clinical trial also indicates that raised level of MDA in patients with affective disorders [44]. Besides, other antidepressant such as fluoxetine has also been reported to reduce the maloanodialdehyde level in restraint animals [45]. Antidepressants drugs have also been reported to elevated antioxidant enzyme defense system particularly superoxide enzyme and catalase activity [46]. These antioxidant enzymes raised the level of oxidative defense against stress.

## Conclusion

Present study highlights the modest activity of SJW against acute restrain stress causes neurobehavioral alterations and oxidative damage. Study provides a hope SJW can be used in the treatment and management of stress conditions.

## Abbreviations

SJW: St. John's Wort; DTNB: dithiobisnitrobenzoic acid; HPA: hypothalamus - pituitary - adrenal axis; ROS: reactive oxygen species; MDA: malondialdehyde.

## Competing interests

The authors declare that they have no competing interests.

## Authors' contributions

RG carried out all behavioral tests in animal. AP carried out all biochemical analysis. AK was involved in all the behavioral, biochemical and data analysis. However, all the three authors equally contributed in data interpretation and drafted the manuscript. All authors read and approved the final manuscript.

## Pre-publication history

The pre-publication history for this paper can be accessed here:

http://www.biomedcentral.com/1472-6882/10/18/prepub
